# MiR-125b regulates epithelial-mesenchymal transition via targeting Sema4C in paclitaxel-resistant breast cancer cells

**DOI:** 10.18632/oncotarget.3065

**Published:** 2014-12-18

**Authors:** Qingling Yang, Yangyang Wang, Xiaohui Lu, Zunlan Zhao, Lihua Zhu, Sulian Chen, Qiong Wu, Changjie Chen, Zhiwei Wang

**Affiliations:** ^1^ Department of Biochemistry and Molecular Biology, Bengbu Medical College, Anhui, China; ^2^ Clinical Testing and Diagnose Experimental Center of Bengbu Medical College, Anhui, China; ^3^ Department of Medical Oncology, First Affiliated Hospital of Bengbu Medical College, Bengbu, Anhui, China; ^4^ Cyrus Tang Hematology Center, Jiangsu Institute of Hematology, Collaborative Innovation Center of Hematology, First Affiliated Hospital of Soochow University, Suzhou, Jiangsu, China

**Keywords:** Paclitaxel, miR-125b, EMT, invasion, breast cancer

## Abstract

Emerging evidence has demonstrated that microRNAs (miRNA) play a critical role in chemotherapy-induced epithelial-mesenchymal transition (EMT) in breast cancer. However, the underlying mechanism of chemotherapy-mediated EMT has not been fully understood. To address this concern, we explored the role of miR-125b in regulation of EMT in stable paclitaxel-resistant (PR) breast cancer cells, namely MCF-7 PR and SKBR3 PR, which have displayed mesenchymal features. Our results illustrated that miR-125b was significantly downregulated in PR cells. Moreover, ectopic expression of miR-125b by its mimics reversed the phenotype of EMT in PR cells. Furthermore, we found that miR-125b governed PR-mediate EMT partly due to governing its target Sema4C. More importantly, overexpression of miR-125b or depletion of Sema4C sensitized PR cells to paclitaxel. These findings suggest that up-regulation of miR-125b or targeting Sema4C could serve as novel approaches to reverse chemotherapy resistance in breast cancers.

## INTRODUCTION

Breast cancer is one of the most common women tumors, which leads to high mortality in the United States [1]. According to the American Cancer Society, 23,270 new breast cancer cases and approximately 40,000 deaths due to this deadly disease are projected to occur in 2014 [2]. It has been documented that chemotherapy is one of the standard therapeutic approaches for the treatment of breast cancer, which has shown to inhibit tumor growth and prolong patient survival [3]. However, some breast cancer patients are resistant to chemotherapy mainly due to intrinsic or acquired resistance, which could lead to failure of chemotherapy and worse treatment outcome [4, 5]. Thus, it is urgent to gain further insight into the molecular mechanism of chemotherapeutic drug resistance, and find a novel strategy for enabling better therapeutic benefits of breast cancer patients.

Increasing evidence has demonstrated that the drug-resistant (DR) cells are associated with epithelial-mesenchymal transition (EMT) in human cancers. It is known that EMT is a complex biological process in which epithelial cells acquire mesenchymal properties. Specifically, cells have decreased expression of the epithelial adhesion marker E-cadherin and gain the increased expression of mesenchymal molecules including Snail, Slug, Vimentin, zinc-finger E-box binding homeobox 1 (ZEB1), and ZEB2, leading to enhanced motility, invasion and metastasis as well as drug resistance [6]. In line with this concept, multiple studies have revealed that chemotherapeutic drug can induce EMT, resulting in drug resistance [7-9]. For example, paclitaxel (also known as taxol) resistant (PR)-epithelial ovarian carcinoma cells displayed EMT features, which could be due to upregulation of phosphatidylinositol 3-kinase (PI3K) [10, 11]. Emerging evidence suggests that elevated chemokine CCL2 (C-C motif ligand 2) was found in PR ovarian cancer cell lines [12]. Moreover, overexpression of several molecules such as human epidermal growth factor receptor 2 (HER2), P-glycoprotein (P-gp), lung resistance-related protein (LRP), glutathione-S-transferase-π (GST-π) was observed in PR breast cancer cells [13]. Our previous study has shown that acquisition of EMT is associated with overexpression of S-phase kinase-associated protein 2 (Skp2) in PR breast cancer cells [14]. Although these studies dissect the PR mechanism, further investigation is required to elucidate the exact molecular basis of PR-induced EMT.

Recently, it has been demonstrated that microRNAs (miRNAs) play a key role in regulation of drug resistance and DR-induced EMT. It is known that miRNAs, a class of small non-coding RNAs of about 19-25 nucleotides in length, negatively regulate gene expression by repressing messenger RNAs (mRNAs) or cleaving mRNAs. It is clear that miRNAs can exert its oncogenic or anti-tumor activities depending on the cellular function of their targets. Fang et al. reported that miR-17-5p promoted chemotherapeutic drug resistance and tumor metastasis through repressing PTEN (phosphatase and tensin homolog deleted on chromosome ten) expression in colorectal cancer [15]. Additionally, miR-25 was found to regulate chemoresistance-associated autophagy in breast cancer cells [16]. Notably, miRNAs were reported to regulate DR-mediated EMT in human breast cancer. For instance, Jiang et al. found that miR-489 governed chemoresistance through regulating Smad3 expression and Smad3 related EMT in breast cancer [17]. Moreover, re-expression of miR-375 sensitized tamoxifen-resistant cells to tamoxifen and partly reversed EMT due to regulation of metadherin in breast cancer [18]. Furthermore, overexpression of miR-200c inhibited the resistance of breast cancer cells to doxorubicin via suppressing E-cadherin-mediated PTEN /Akt signaling pathway [19]. Consistently, restoration of miR-200c increased trastuzumab sensitivity and inhibited invasiveness of breast cancer cells through regulating EMT and targeting ZEB1 and zinc finger gene 217 (ZNF217) [20]. In support of the role of miR-200 family in drug resistance, it has been reported that reduced miR-200b and miR-200c expression contributed to endocrine resistance in breast cancer cells [21]. These reports unraveled the crucial roles of miRNAs in drug resistance-mediated EMT; however, whether miR-125b has a potential role in PR-induced EMT remains largely unknown.

In the present study, we explored the role of miR-125b in controlling PR-mediated EMT in breast cancer cells. We further determine whether Sema4C, one of miR-125b targets, was involved in PR-induced EMT. We identified that miR-125b was down-regulated in PR cells and overexpression of miR-125b reversed the mesenchymal features in breast cancer cells. Notably, ectopic expression of miR-125b or depletion of its target Sema4C sensitized PR cells to paclitaxel. Our results revealed that re-expression of miR-125b or inhibition of Sema4C could be potential therapeutic approaches for treating PR breast cancer.

## RESULTS

### Downregulation of miR-125b is observed in PR cells

**Figure 1:**
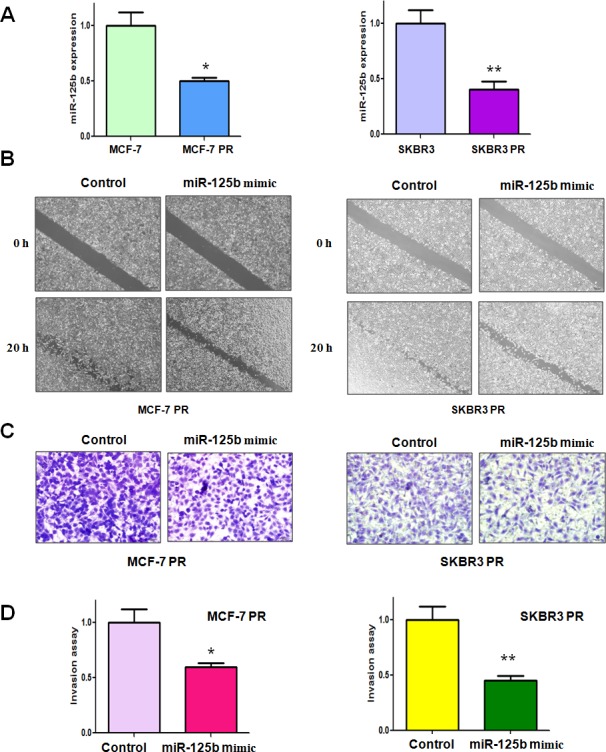
miR-125b mimic inhibited motility and invasion in paclitaxel-resistant (PR) breast cancer cells A, TaqMan miRNA assay was conducted to determine the expression of miR-125b in parental and PR cells. * P<0.05, **P<0.01 PR vs control. B, Wound healing assays were used to detect the motility in PR cells transfected with miR-125b mimic. C, Invasion assay were conducted in PR cells transfected with miR-125b mimic. D, Quantitative results are illustrated for panel C. * P<0.05, **P<0.01 vs control.

It has been demonstrated that miR-125b is critically involved in regulating EMT in human cancer [22]. To determine whether miR-125b plays a pivotal role in PR-induced EMT in breast cancer cells, we measured the expression of miR-125b in PR cells and their parental cells. Our results revealed that miR-125b was significantly downregulated in PR cells (Figure 1A). This finding indicated that downregulation of miR-125b could contribute to PR-induced EMT.

### Overexpression of miR-125b inhibits cell motility and invasion in PR cells

To further validate the role of miR-125b in regulating EMT, we determined the cell migration and invasion in PR cells after miR-125 mimic treatment by wound healing assay and invasion assay, respectively. Our wound healing assay showed that miR-125b mimic inhibited the cell migration in both MCF-7 PR and SKBR3 PR cells (Figure 1B). In line with this result, we found that miR-125b mimic suppressed the cell invasion in PR cells (Figure 1C, 1D). Our results suggest that miR-125b could play a critical role in governing PR-mediate EMT.

### Overexpression of miR-125b reverses EMT in PR Cells

**Figure 2:**
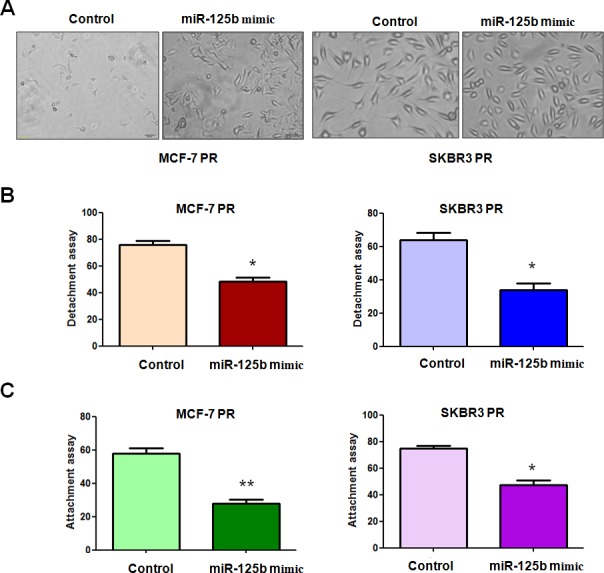
miR-125b mimc reduced the capacity of attachment and detachment in PR breast cancer cells A, Cell morphology was taken by microscopy in PR cells treated with miR-125b mimic. B, Cell detachment assays were conducted in PR cells transfected with miR-125b mimic. * P<0.05 vs control. C, Cell attachment assays were measured in PR cells transfected with miR-125b mimic. * P<0.05, **P<0.01 vs control.

**Figure 3:**
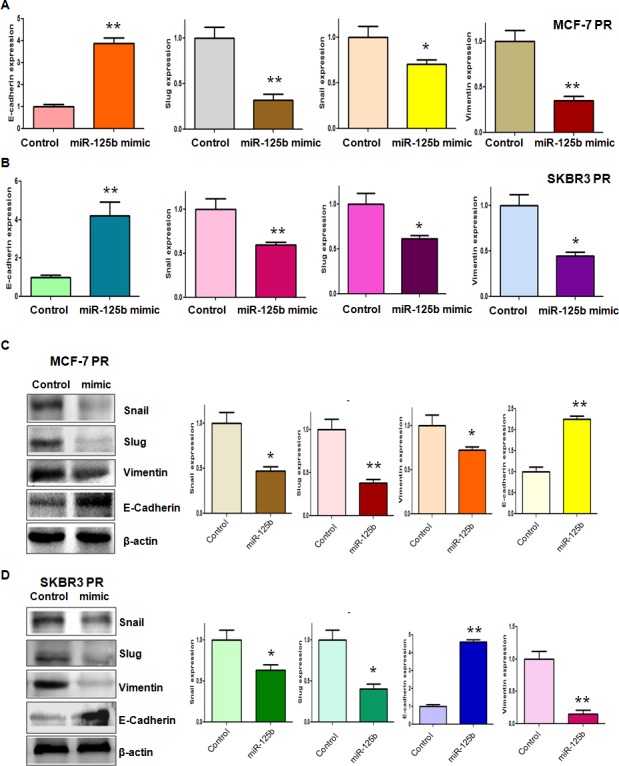
miR-125b mimic regulated the expression of EMT markers in PR breast cancer cells A-B, Real-time RT-PCR analysis was performed to detect the mRNA levels of EMT markers in MCF-7 PR (A) and SKBR3 PR (B) cells after miR-125b mimic treatment. * P<0.05, **P<0.01 vs control. C-D, Left panel: Western blotting analysis was performed to detect the expression of EMT markers in MCF-7 PR (C) and SKBR3 PR (D) cells after miR-125b mimic treatment. Right panel: Quantitative results are illustrated for left panel. * P<0.05, **P<0.01 vs control.

Our previous study has shown that PR cells have mesenchymal features [14]. To further explore whether ectopic expression of miR-125b could reverse the mesenchymal characteristics in PR cells, we transfected miR-125b mimic into MCF-7 PR and SKBR3 PR cells. We observed that miR-125b mimic treatment led to reversal of EMT in PR cells, which have changed from elongated, fibroblastoid morphology to a rounded shape (Figure 2A). Consistently, miR-125b mimic treatment reduced the capacity of detachment and attachment in PR cells (Figure 2B, 2C). In support of this notion, we measured the expression of EMT markers in PR cells treated with miR-125b mimic by real-time RT-PCR and Western blotting analysis. We found that miR-125b mimic transfection caused the higher expression of E-cadherin and lower expression of mesenchymal markers such as Snail, Slug and Vimentin at both mRNA (Figure 3A, 3B) and protein levels (Figure 3C, 3D) in PR cells. Our results revealed that reduction of miR-125b could be responsible for PR-induced EMT in breast cancer cells.

### MiR-125b regulates EMT by targeting Sema4C in PR cells

**Figure 4:**
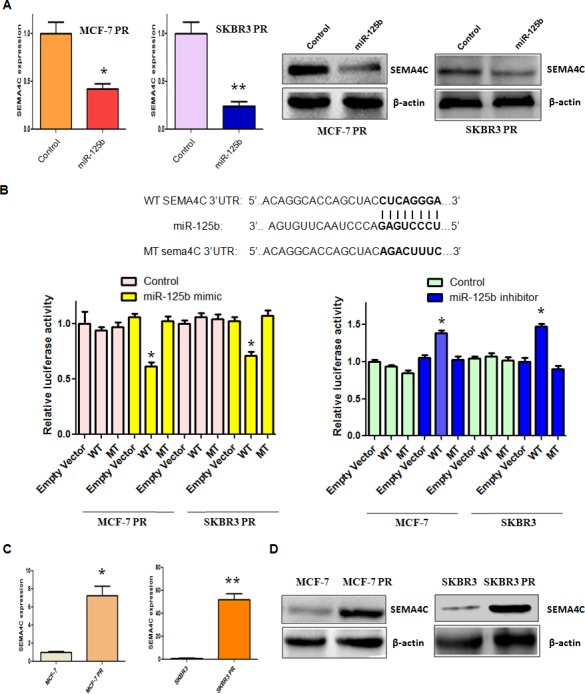
miR-125b targeted Sema4C expression A, Real-time RT-PCR assay (Left panel) and Western blotting analysis (Right panel) were performed to detect the expression of Sema4C at the mRNA and protein levels, respectively, in PR cells treated with miR-125b mimic. * P<0.05, **P<0.01 vs control. B, Top panel: Sequences of wild-type and mutant target sites for miR-125b in Sema4C are shown. Bottom panel: Luciferase reporter assays were performed to identify the binding of miR-125b to Sema4C 3′-UTR. C, Real-time RT-PCR assay was performed to detect the mRNA level of Sema4C in PR cells. * P<0.05, **P<0.01 vs control. D, Western blotting analysis was conducted to measure the expression of Sema4C in PR and their parental cells.

It has been well known that miRNA exerts its function via binding to the 3′-UTR (untranslated region) of target genes through partial sequence homology. Therefore, to further investigate the role of miR-125b in controlling PR-mediated EMT, we used two target prediction programs, TargetScan and miRanda, to screen the potential targets of miR-125b. Our analysis predicted that Sema4C could be a potential miR-125b target. To confirm this concept, we measured the expression of Sema4C at mRNA and protein levels by real-time RT-PCR and Western blotting analysis, respectively. We found that miR-125b overexpression decreased Sema4C expression at both mRNA and protein levels in MCF-7 PR and SKBR3 PR cells (Figure 4A). To further validate whether Sema4C is a direct and specific target of miR-125b, miR-125b mimic and Sema4C 3′-UTR wild type or 3′-UTR mutated luciferase reporter were transfected into MCF-7 PR and SKBR3 PR cells. We found that miR-125b mimic decreased Sema4C 3′-UTR wild-type, but not Sema4C 3′-UTR mutation in which the binding sites for miR-125b were mutated, reporter activity (Figure 4B). Moreover, we observed that miR-125b inhibitor increased Sema4C 3′-UTR wild-type, but not Sema4C mutation, in MCF-7 and SKBR3 cells (Figure 4B). Taken together, these findings suggest that miR-125b specifically targets the 3′-UTR of Sema4C and subsequently inhibits its expression. Consistently, we found the high expression of Sema4C in MCF-7 PR and SKBR3 PR cells compared with their parental cells (Figure 4C, 4D).

### Depletion of Sema4C inhibits motility and invasion in PR cells

**Figure 5:**
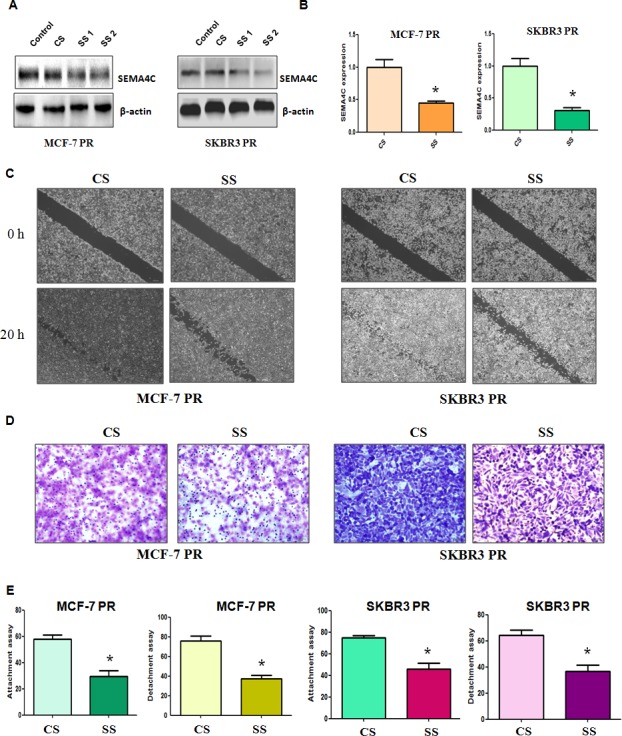
Depletion of Sema4C inhibited motility and invasion in PR cells A, Western blotting analysis was performed to detect the expression of Sema4C in PR cells treated with Sema4C siRNA. CS: control siRNA; SS: Sema4C siRNA. B, Real-time RT-PCR assay was used to measure the Sema4C mRNA level in PR cells treated with Sema4C siRNA. * P<0.05 vs control. C, Wound healing assays were performed in PR cells transfected with Sema4C siRNA. D, Invasion assay was performed in PR cells transfected with Sema4C siRNA. E, Cell detachment and attachment assays were performed in PR cells transfected with Sema4C siRNA. * P<0.05 vs control.

To further determine whether Sema4C is involved in PR-mediated EMT, we measured the cell motility and invasion capacities in PR cells transfected with Sema4C siRNA. We found that both Sema4C siRNA1 and its siRNA2 transfection markedly inhibited Sema4C expression in MCF-7 PR and SKBR3 PR cells (Figure 5A). We used Sema4C siRNA2 for our following studies. Sema4C RNA level was also significantly downregulated by Sema4C siRNA treatment (Figure 5B). Moreover, our wound healing assay showed that depletion of Sema4C retarded cell motility in PR cells (Figure 5C). Consistent with this result, Sema4C siRNA transfection inhibited the cell invasion in MCF-7 PR and SKBR3 PR cells (Figure 5D). Notably, Sema4C siRNA treatment reduced the capacity of cell detachment and attachment in PR cells (Figure 5E).

### Depletion of Sema4C regulates expression of EMT markers

**Figure 6:**
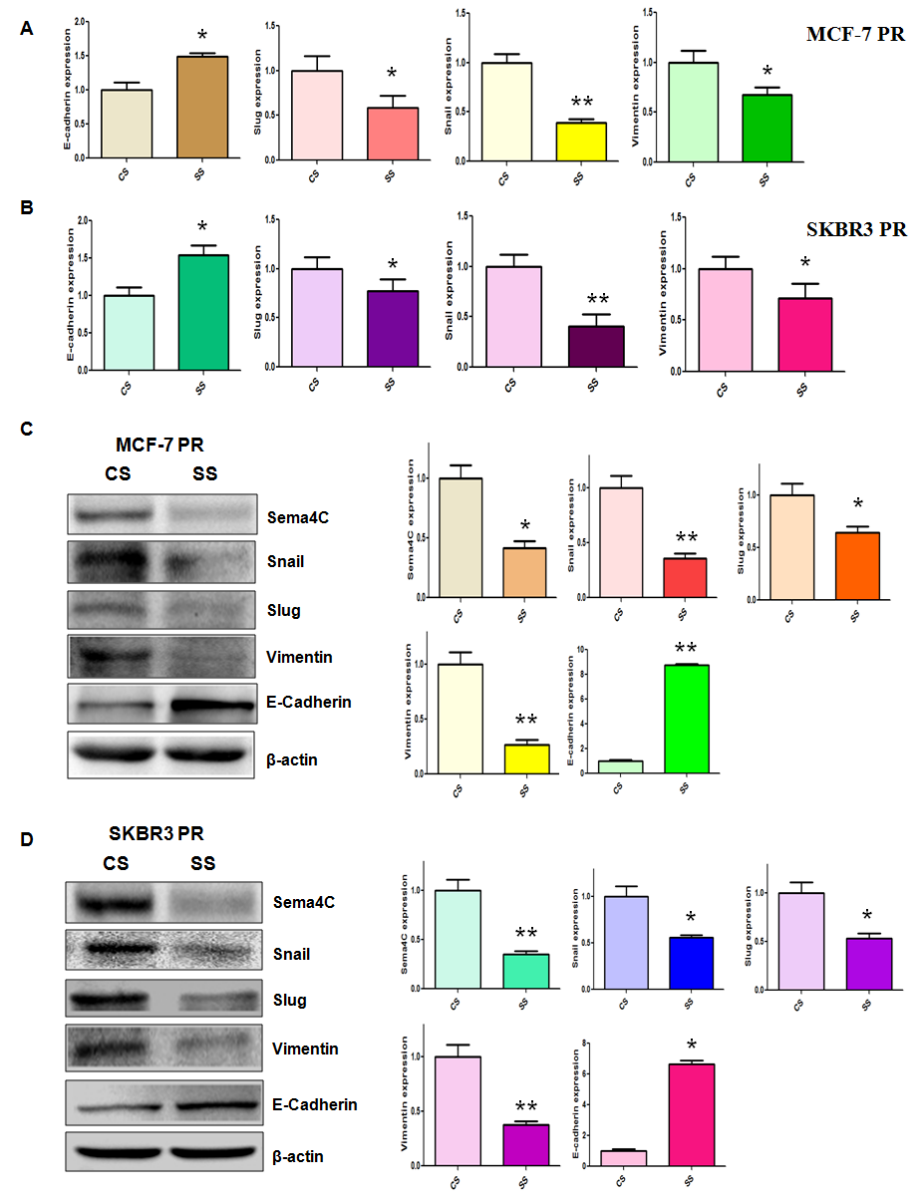
Depletion of Sema4C regulated the expression of EMT markers in PR cells A-B, Real-time RT-PCR was performed to quantify mRNA expression of EMT markers in PR cells transfected with Sema4C siRNA. CS: control siRNA; SS: Sema4C siRNA. *, P<0.05, **P<0.01 compared with control siRNA. C-D, Left panel: Western blotting analysis was used to detect the expression of EMT markers in PR cells transfected with Sema4C siRNA. Right panel: Quantitative results are illustrated for left panel. CS: control siRNA; SS: Sema4C siRNA. *, P<0.05, **P<0.01 compared with control siRNA.

To further dissect whether down-regulation of Sema4C governs the expression of EMT markers in PR cells, we detected the mRNA and protein levels of EMT markers in PR cells after Sema4C siRNA transfection. Our real-time RT-PCR assay and Western blotting analysis showed that depletion of Sema4C increased the E-cadherin mRNA and protein levels, but decreased the expression of mesenchymal markers including Snail, Slug, and Vimentin (Figure 6A-6D). Taken together, these results indicated that Sema4C plays a critical role in regulating PR-induced EMT in breast cancer cells.

### Up-regulation of miR-125b or depletion of Sema4C enhances PR cells to paclitaxel sensitivity

**Figure 7:**
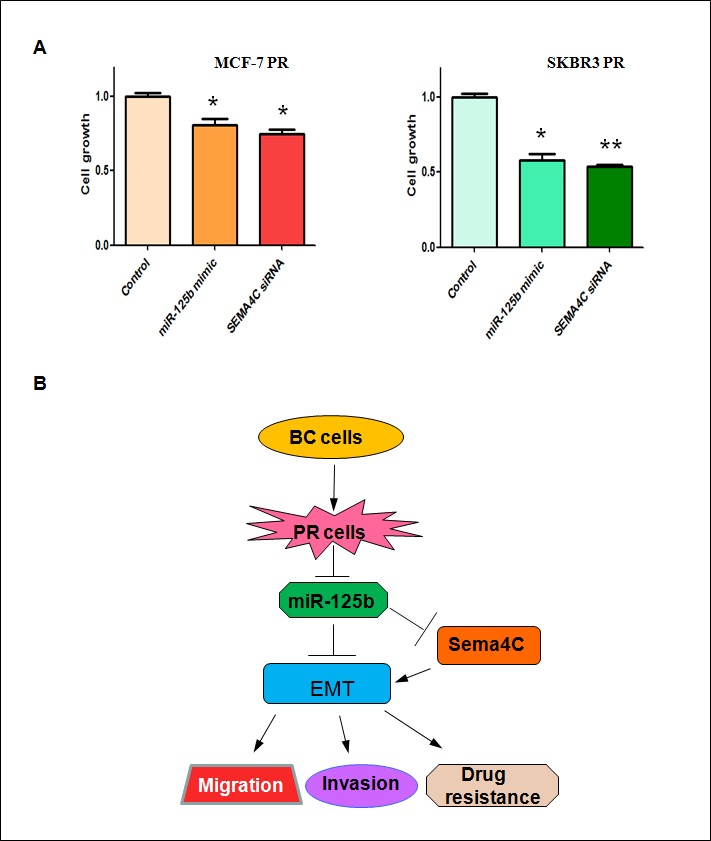
Up-regulation of miR-125b or depletion of Sema4C enhanced PR cells to paclitaxel sensitivity A, SRB assay was performed in PR cells transfected with miR-125b mimic or Sema4C siRNA and then treated with 10μg/ml paclitaxel for 72 hours. * P<0.05, **P<0.01 vs control. B, A model for miR-125b in paclitaxel resistance cells is proposed. BC: breast cancer; PR: paclitaxel resistant; EMT: epithelial-mesenchymal transition.

To determine whether up-regulation of miR-125b or depletion of Sema4C enhances PR cells to paclitaxel sensitivity, we conducted SRB assay in PR cells treated with miR-125b mimic or Sema4C siRNA. We found that overexpression of miR-125b or downregulation of Sema4C significantly promoted cell growth inhibition induced by 10μg/ml paclitaxel in MCF-7 PR and SKBR3 PR cells (Figure 7A). These findings demonstrated that PR cells with miR-125b mimic or Sema4C siRNA treatment were significantly more sensitive to paclitaxel-induced cell growth inhibition.

## DISCUSSION

Dysregulation of miR-125b has been identified to play an important role in the initiation and progression of human malignancies including breast cancer [23]. For instance, miR-125b was downregulated in breast cancer primary tumors compared with normal breast samples [24-30]. Moreover, overexpression of miR-125b inhibited cell growth and reduced migration and invasion in breast cancer cells through suppression of ERBB2 (erythroblastic leukemia viral oncogene homolog 2) and ERBB3 in breast cancer cells [31]. Additionally, downregulation of miR-125b increased breast cancer cell growth through upregulation of Mucin1, whereas upregulation of miR-125b enhanced the apoptosis response of breast cancer cells to cisplatin treatment [32]. Zhang et al. reported that miR-125b is methylated and functions as a tumor suppressor through targeting the ETS1 proto-oncogene in human invasive breast cancer [33]. In light of these findings, one study showed that overexpression of miR-125b retarded motility and migration behaviors via targeting ARID3B (T-rich interactive domain 3B) in breast cancer cells [34]. Recently, miR-125b was validated to target erythropoietin and its receptor and their expression is associated with metastatic potential in breast cancer [28]. In consistent with these reports, miR-125b was found to exert its anti-tumor activity via regulating ENPEP (aminopeptidase A), CK2-α (casein kinase 2 alpha), CCNJ (cyclin J) and MEGF9 (multiple EGF-like-domains 9) in breast cancer [29]. Furthermore, the decreased miR-125b levels in breast cancer tissues led to high expression of its target CYP24 (Vitamin D3 hydroxylase), which could cause tumorigenesis [35]. However, Fang et al. found that miR-125b could induce metastasis through targeting STARD13 (StAR-related lipid transfer domain containing 13) in breast cancer cells [36], suggesting that further investigation is necessary to explore the function of miR-125b in tumor metastasis. In the current study, we observed that overexpression of miR-125b inhibited cell migration and invasion through targeting Sema4C in breast cancer cells.

Several studies have demonstrated that Sema4C, a member of the semaphorin family, plays a crucial role in regulating EMT. For example, Sema4C induced EMT through suppression of E-cadherin and induction of Vimentin in HK2 cells [37]. In line with this, knockdown of Sema4C reversed TGF-β1 (transforming growth factor, beta 1)-mediated EMT through suppressing phosphorylation of p38 MAPK (mitogen-activated protein kinase), whereas upregulation of Sema4C promoted EMT via activation of p38 MAPK phosphorylation in human proximal tubular epithelial cells [38]. Moreover, high expression of Sema4C has been observed in esophageal cancer, gastric cancer and rectal cancer and is correlated with lymphatic metastasis [39]. In support of the role of Sema4C in controlling EMT, our results showed that it has high expression of Sema4C in EMT-type PR cells. More importantly, depletion of Sema4C regulated expression of EMT markers and inhibited motility and invasion in PR cells. Notably, we identified Sema4C as a target of miR-125b, indicating that miR-125b exerts its function partly due to governing Sema4C expression.

Accumulating evidence has suggested that miR-125b was critically involved in drug resistance. It has been reported that overexpression of miR-125b sensitized cells to 5-fluorouracil by regulation of hexokinase II and inhibition of glycolysis in human hepatocellular carcinoma cells [40]. Moreover, miR-125b was reported to promote leukemia cell resistance to daunorubicin through inhibiting expression of G protein-coupled receptor kinase 2 and apoptosis [41]. Interestingly, studies have identified that inhibition of miR-125b enhanced the chemosensitivity of glioblastoma stem cells to temozolomide through regulating Bak1 (Bcl-2 antagonist killer 1) and PIAS3 (protein inhibitor of activated signal transducer and activator of transcription) [42-44]. In addition, it has been shown that miR-125b was overexpression in vincristine-resistant acute lymphoblastic leukemia [45]. Consistently, studies have revealed that overexpression of miR-125b increased drug resistance in pediatric acute promyelocytic leukemia [46], Ewing sarcoma/primitive neuroectodermal tumor [47], and cutaneous T-cell lymphomas [48]. To further determine the role of miR-125b in drug resistance in breast cancer, we measured the expression of miR-125b in PR cells and observed down-regulation of miR-125b in PR cells. Remarkably, we found that overexpression of miR-125b sensitized PR cells to paclitaxel in breast cancer cells. Surprisingly, one study showed that miR-125b was downregulated in PR ovarian cancer cells, but upregulated in cisplatin-resistant ovarian cancer cells [49], suggesting that miR-125b could play an opposite role in different drug resistant cell lines. However, it has been considered that miR-125b upregulation dictates its oncogenic characteristics, while miR-125b downregulation leads to the loss of tumor suppressor functions [23]. Without a doubt, in-depth investigation is required to explore the function of miR-125b in different tumor entities.

Due to the important role of miR-125b involved in drug resistance and EMT, targeting miR-125b could be a novel strategy for treating human cancers (Figure 7B). To this end, several groups have discovered multiple agents that regulated the miR-125b expression. We have found that arsenic trioxide could regulate the expression of miR-125b in human glioma [50]. A short hairpin-looped DNA analogous oligodeoxynucleotide (ODN) to miR-125b has been shown to inhibit C-Raf (C-rapidly accelerated fibrosarcoma) expression, proliferation, and survival of breast cancer cells [51]. Wang et al. found that the class I HDAC (histone deacetylase) inhibitor entinostat induced miR-125b expression and subsequently downregulated ERBB2 and ERBB3 expression, leading to cell apoptosis in breast cancer cells [52]. However, it is pivotal to develop the specific miR-125b inhibitor or activator for the treatment of human cancer. Taken together, activation of miR-125b or inactivation of Sema4C may be a useful strategy to reverse chemotherapy resistance in breast cancer.

## MATERIALS AND METHODS

### Cell culture

Human breast cancer cell lines MCF-7, MCF-7 PR, SKBR3 and SKBR3 PR were cultured in RPMI 1600 (Invitrogen, Carlsbad, CA) supplemented with 10% fetal bovine serum (FBS), and maintained in a humidified 5% CO_2_ incubator at 37°C. MCF-7 PR and SKBR3 PR cells displayed resistance to cell growth inhibition of 10μg/ml paclitaxel [14]. The resistant cells were maintained in culture medium with 10μg/ml paclitaxel.

### Reagents and antibodies

Tumor Invasion Assay Kit was bought from BD Biosciences (Bedford, MA, USA). Sulforhodamine B (SRB) was purchased from Sigma (St. Louis, Mo). Primary antibodies against Vimentin, Snail, Slug, E-cadherin, Sema4C, and β-actin were bought from Santa Cruz Biotechnology (Santa Cruz, CA).

### Cell proliferation assay

The transfected cells (5 ×10^3^) were seeded in each well of the 96-well plates for overnight incubation. Then, the cells were treated with 10μg/ml paclitaxel for 72 hours. After the culture medium was removed, 200μl of 10% trichloroacetic acid was added and incubated at 4 °C for 1 h. The plates were flicked, washed, and stained with 100μl SRB for 30 min at 37 °C. The unbound dye was removed and air-dried. Then, 150μl of 10 mmol/L Tris was added to the plates to solubilise the dye. The absorbance was measured by a microplate reader at a wavelength of 515nm.

### Wound healing assay

The cells were seeded in 6-well plate until the cells reached to 90% confluency. The confluent monolayers were scratched by a 200 μl pipette tip to generate the wound. The debris and floating cells were removed though washing with PBS. The cells were cultured for 20 hours to allow wound healing. The photographic images were taken at 0 hour and 20 hours.

### Transwell invasion assays

The invasive activity of cells was measured using Transwell inserts precoated with Matrigel as described earlier [53]. Briefly, cells were added to the upper chamber of the inserts. Cell culture medium with 10% FBS was added to the lower chamber. The cells were allowed to invade for 20 hours at 37°C. After removing cells on the upper side of the transwell, the invading cells on the underside were fixed with 4% paraformaldehyde, and stained with Giemsa solution. The stained invaded cells were photographed under a microscope.

### Reverse transcription-PCR analysis for gene expression

The total RNA was isolated with Trizol (Invitrogen, Carlsbad, CA) according to the manufacturer's protocols. The SYBR green RT-PCR assay (Takara, Dalian, China) were described previously [50]. The primers used in PCR reaction are below: Sema4C, forward primer: 5′-ACC TTG TGC CGC GTA AGA CAG-3′; and reverse primer: 5′-CGT CAG CGT CAG TGT CAG GAA-3′; GAPDH, forward primer: 5′-CAG CCT CAA GAT CAT CAG CA-3′ and reverse primer: 5′-TGT GGT CAT GAG TCC TTC CA-3′. The expression of EMT markers including E-cadherin, Snail, Slug, and Vimentin was determined as described previously [14].

### Western blotting analysis

Cells were lysed with RIPA buffer supplemented with protease inhibitors. The protein concentrations were measured using the Bio-Rad protein assay kit (Bio-Rad Laboratories, CA). The proteins were resolved through 8% SDS-polyacrylamide gel electrophoresis and then electrotransferred to polyvinylidene difluoride membranes. These membranes were immunoblotted with indicated antibodies, and then with peroxidase-conjugated secondary antibody for Western blotting as described previously [54].

### miRNA real-time RT-PCR

The miRNA RT-PCR was used to detect the alterations of miR-125b expression in breast cancer cells as described previously [50]. Briefly, 10 ng of total RNA was reversed transcribed into cDNA using TaqMan miRNA hsa-miR-125b-specific primers (Applied Biosystems). Then real-time PCR was performed using a TaqMan MicroRNA Reverse Transcription Kit (Applied Biosystems). RNA *U6* was carried out as endogenous control in each sample. The relative expression was analyzed using the comparative *C*t method.

### Transfection

Cells were seeded in six-well plates and transfected with Sema4C siRNA, or control siRNA using Lipofectamine 2000 as described before [53]. The sequences used for Sema4C siRNA are as followed: Sema4C siRNA1, Forward oligo, 5′-GGA GCA UGG AGA GUU UGA ATT-3′; Reverse oligo, 5′-UUC AAA CUC UCC AUG CUC CTT-3′; Sema4C siRNA 2, Forward oligo, 5′-GGC UCG UGC AUU AAC AAC UTT-3′; Reverse oligo, 5′-AGU UGU UAA UGC ACG AGC CTT-3′. The non-targeting control siRNA, Forward oligo, 5′-UUC UCC GAA CGU GUC ACG UTT-3′; Reverse oligo, 5′ ACG UGA CAC GUU CGG AGA ATT-3′. After the transfection, the cells were used for further analysis as described under the results section.

### MiRNA-125b mimic transfection

The cells were seeded in six-well plates and transfected with miR-125b mimic or the nonspecific control (GenePharma, shanghai, china) using lipofectamine RNAiMAX reagent (Invitrogen) following the manufacture's protocol. MiR-125b mimic: Sense 5′-UCC CUG AGA CCC UAA CUU GUG A-3′; antisense 5′-ACA AGU UAG GGU CUC AGG GAU U-3′. The cells were subjected to further analysis as presented under the results section.

### MiRNA-125b inhibitor transfection

The cells were seeded in 24-well plates and transfected with miR-125b inhibitor or the nonspecific control using DharmaFect Transfection Reagent (Dharmacon, CO) following the manufacture's protocol. MiR-125b inhibitor: 5′-UCA CAA GUU AGG GUC UCA GGG A-3′. The cells were subjected to analysis by luciferase reporter assay.

### Luciferase reporter assay

The miR-125b response element (wide type or mutated) in the 3′-UTR of Sema4C was cloned into pMIR-REPORT plasmid downstream of luciferase reporter gene. Cells seeded into 24-well plates were cotransfected with miR-125b and luciferase reporter constructs containing WT or MT Sema4C 3′-UTR. We used a luciferase assay kit to analyze luciferase activities.

### Cell attachment and detachment assay

Cell attachment and detachment assays were conducted as described previously [55]. Briefly, for attachment assay, non-attached cells were washed after the cells were seeded in 24-well plate for one hour. The attached cells were trypsinized and counted. For detachment assay, the cells were detached with trypsin for 3 minutes and counted after they were seeded for overnight. The attached cells were also counted after trypsinization. The data were quantified as a percentage of the attached/detached cells to total cells.

### Statistical Analysis

Statistical comparisons between two different groups were determined by Student's *t*-test using GraphPad Prism 4.0 (Graph pad Software, La Jolla, CA). The results were presented as means ± SEM. P<0.05 was considered statistically significant.
